# Detecting SARS-CoV-2 Orf3a and E ion channel activity in COVID-19 blood samples

**DOI:** 10.1017/cts.2021.856

**Published:** 2021-09-14

**Authors:** Han-Gang Yu, Gina Sizemore, Katy Smoot, Peter Perrotta

**Affiliations:** 1 Department of Physiology and Pharmacology, School of Medicine, West Virginia University, Morgantown, WV, USA; 2 EZCARE Walk-In Medical Center, Moorefield, WV, USA; 3 Department of Pathology, Anatomy and Laboratory Medicine, School of Medicine, West Virginia University, Morgantown, WV, USA

**Keywords:** COVID-19, SARS-CoV-2, channel activity detection, blood

## Abstract

**Background:**

SARS-CoV-2 has been found in the heart of COVID-19 patients. It is unclear how the virus passes from the upper respiratory tract to the myocardium. We hypothesized that SARS-CoV-2 is present in the blood of COVID-19 infected patients, spreading to other organs such as heart.

**Methods:**

We targeted two viroporins, Orf3a and E, in SARS-CoV-2. Orf3a and E form non-voltage-gated ion channels. A combined fluorescence potassium ion assay with three channel modulators (4-aminopyridine, emodin-Orf3a channel blocker, and gliclazide-E channel blocker) was developed to detect SARS-CoV-2 Orf3a/E channel activity. In blood samples, we subtracted the fluorescence signals in the absence and presence of emodin/gliclazide to detect Orf3a and E channel activity.

**Results:**

In lentivirus-spiked samples, we detected significant channel activity of Orf3a/E based on increase in fluorescence induced by 4-aminopyridine, and this increase in fluorescence was inhibited by emodin and gliclazide. In 18 antigen/PCR-positive samples, our test results found 15 are positive, demonstrating 83.3% concordance. In 24 antigen/PCR-negative samples, our test results found 21 are negative, showing 87.5% concordance.

**Conclusions:**

We developed a cell-free test that can detect Orf3a/E channel activity of SARS-CoV-2 in blood samples from COVID-19-infected individuals, confirming a hypothesis that the virus spreads to the heart via blood circulation.

## Introduction

COVID-19 infection is caused by the SARS-CoV-2 virus. Various testing methods are used to detect SARS-CoV-2 viral RNA, viral antigen, and antibodies formed in response to COVID-19 infection or vaccination [8-10]. COVID-19 infection is identified by highly sensitive PCR tests that detect the presence of viral RNA in upper respiratory (e.g., nasal, nasopharyngeal, oral) and saliva samples. Antigen test also identifies current infection by using antibodies specific to SARS-CoV-2 viral proteins including spike and nucleocapsid proteins. Antibody tests measure immune-reactive antibodies that develop following more recent (IgM isotype) or past (IgG isotype) infection.

SARS-CoV-2 *RNA* has been detected by RT-PCR in up to 76% of blood or serum samples collected from patients with COVID-19 infection [2]. SARS-CoV-2 has not been detected in blood using antigen-based tests according to the CDC Interim Guidance for Antigen Testing for SARS-CoV-2 [9]. To the best of our knowledge, SARS-CoV-2 *viral proteins* have not been reported in blood samples of COVID-19 patients by three testing methods.

In addition to primary infection in the respiratory tract, SARS-CoV-2 can cause damage in multiple organs including the heart. The initial hypothesis presumed that virus-mediated inflammation was the primary cause of cardiac dysfunctions [4,15]. Recent reports provided evidence for the presence of SARS-CoV-2 in the myocardium of COVID-19 patients [27] and in heart tissues of COVID-19 autopsy cases [3,18]. Direct viral entry into cardiac myocytes is the primary mechanism of myocardial infection [3]. This is not surprising since the SARS-CoV-2 binding receptor, angiotensin-converting enzyme 2 (ACE2), was initially discovered in [13,28] and highly expressed by the heart [11,19]. However, it is unclear how the virus passes from the upper respiratory tract to the myocardium; one likely explanation is via blood circulation.

SARS-CoV-2 contains two viroporins, Orf3a and E, both of which can form non-voltage-gated cation channels [22,25,26]. We report here that the protein motifs that determine the combined channel activity of Orf3a and E are conserved and specific to SARS-CoV-1 and SARS-CoV-2. Combining a fluorescence ion assay with the channel modulators, we developed a test that can detect channel activity of Orf3a/E in blood samples (whole blood, plasma, serum) of COVID-19 patients in a high-throughput manner.

## Methods

### COVID-19 Patient Blood Samples

Use of blood samples from COVID-19 patients was approved by the West Virginia University Institutional Review Board. Research was discussed with each patient, and if the patient consented to the research project, whole blood was drawn by standard venipuncture into one tube, containing EDTA for anticoagulation. Plasma/sera samples were stored in tube without EDTA. A total of 42 samples were collected: 28 whole blood samples and 14 plasma/serum samples.

### *Fluorescence K* ^*+*^ *Assay and Orf3a/E Channel Activity Detection*

A fluorescence-based potassium ion channel assay utilizes the ability of thallium (Tl^+^) to permeate K^+^ channels [30]. Once the K^+^ channels are open, Tl^+^ in the extracellular solution flows down its concentration gradient into the cells via K^+^ channels. Inside the cells, Tl^+^ binds to and activates a fluorogenic indicator dye preloaded into the cells, resulting in a dramatic increase in fluorescence signal. This technique allows rapid determination of K^+^ channel activity in a high-throughput manner [24]. We used a commercial kit (FluxOR Potassium ion channel assay, cat#: F10016, Thermo Fisher Scientific) according to the manufacturer’s instructions to study fluorescence detection of Orf3a/E channel activity.

Samples of 10 μL each were added to a 96-well plate. Each sample was duplicated in two separate wells: the first replicate contained only assay solution to establish the baseline signal and the second contained both assay solution and Orf3a/E channel blockers. Fluorescence of each sample was measured 3–6 times using a BioTek Synergy H4 Hybrid Microplate Reader.

### Ion Channel Modulators

Emodin, gliclazide, and 4-aminopyridine (4-AP) were purchased from Sigma. Stock solution (50 mm) was prepared in DMSO. Approximately 0.5 mm emodin and gliclazide were used in ∼100 uL solution. DMSO at ∼1–2% of test solution had no effects on test results.

### Lentivirus Containing Orf3a and E

Lentivirus containing SARS-CoV-2 Orf3a and lentivirus containing E were obtained from Addgene donated by Nevan Krogen lab: pLVX-EF1alpha-SARS-CoV-2-orf3a-2xStrep-IRES-Puro was a gift from Nevan Krogan (viral prep # 141383-LV; http://n2t.net/addgene:141383-LV ; RRID:Addgene_141383) [17]. pLVX-EF1alpha-SARS-CoV-2-E-2xStrep-IRES-Puro was a gift from Nevan Krogan (viral prep # 141385-LV; http://n2t.net/addgene:141385-LV; RRID:Addgene_141385) [17]. We used 1 μL lentivirus with the titer ≥ 1x10^6^ TU/mL, containing ∼1000 functional lentiviral particles in the experiments (TU: transduction unit).

### Antigen Testing

Antigen testing was performed in the clinical laboratory using the CareStart Rapid Diagnostic Test for the Detection of SARS-CoV-2 Antigen (Access Bio, Somerset, NJ). Briefly, the nasopharyngeal swab is removed from pouch and the swab is introduced to the nasal passage until it reaches the posterior nasopharynx. The swab is rotated 3–5 times over the posterior nasopharynx and then removed from the nostril with rotation to sample the anterior nares. In the laboratory, the seal is removed from the extraction vial containing the extraction buffer. The swab is placed in the extraction vial and rotated vigorously 5 times. The extraction vial is then squeezed while the swab is removed by rotating against the sides of the extraction vial to remove any excess fluid from the swab, and a cap is placed on the extraction vial. The sample is mixed by tapping the bottom of the extraction tube, inverted, and 3 drops are squeezed into the sample well. Results are read at ten minutes.

A red control line will appear at top of well by the letter “C.” If the test is positive, a blue line will appear below the red line and across from the letter “T.” Positive result reveals red and blue lines. Negative results reveal only a single red line. A result with a blue line without a red line is invalid and test is repeated.

### Data Analysis

Fluorescence data were collected using Gen5 2.0 microplate reader software (BioTek), processed in Excel, and analyzed and plotted using GraphPad Prism 8.

## Results

### Characterization of COVID-19 Blood Samples

Table 1 summarizes the characterization of blood samples from COVID-19 patients. Antigen testing was performed for whole blood samples at the clinic site to identify current infection of the patients. PCR tests were performed for plasma/serum samples to inform infection as current.

**Table 1.** Characterization of COVID-19 blood samples

Whole blood			
Patient #	Antigen test	Our test	Note
1*	Positive	Positive	Onset
1*	Negative	Negative	Recovered
2	Negative	Negative	
3	Negative	Negative	
4	Negative	Negative	
5	Positive	Positive	
6	Positive	Positive	
7	Negative	Negative	
8	Negative	Negative	
9	Positive	Positive	
10	Negative	Negative	
11**	*Positive*	*Negative*	*PCR negative (so, deemed negative)*
12	Negative	Negative	
13	Positive	Positive	
14**	*positive*	*negative*	*PCR result unavailable*
15	Positive	Positive	
16	Positive	Positive	
17	Positive	Positive	
18	*positive*	*negative*	*PCR result unavailable*
19	Negative	Negative	
20	Negative	Negative	
21	Negative	Negative	
22	Negative	Negative	
23	Negative	Negative	
24	Negative	Negative	
25	*positive*	*negative*	*PCR result unavailable*
26	Negative	Negative	
27	*positive*	*negative*	*PCR result unavailable*
28	Negative	Negative	
*from same patient with mild symptoms. **discrepancy between antigen and our tests. Prevalence of COVID-19-positive cases collected in the point-of-care clinic: 14/29 = 48.3%.			

Plasma/serum samples			
Patient#	PCR (swab)	Our test	Notes
1*	Positive	Positive	**√**
2	Negative	Negative	**√**
3	Positive	Positive	**√**
4	Negative	Negative	**√**
5**	*Negative*	*Positive*	X
6**	*Negative*	*Positive*	X
7	Positive	Positive	**√**
8	Positive	Positive	**√**
9	Negative	Negative	**√**
10	Positive	Positive	**√**
11	Positive	Positive	**√**
12	Negative	Negative	**√**
13**	*Negative*	*Positive*	X
14	Positive	Positive	**√**

* plasma, #2-#14 are sera.

** discrepancy. Prevalence of COVID-19-positive cases collected in the hospital: 7/14 = 50%.

### Conservation of E/Orf3a Motifs that form Ion Channels

**Fig. 1.**
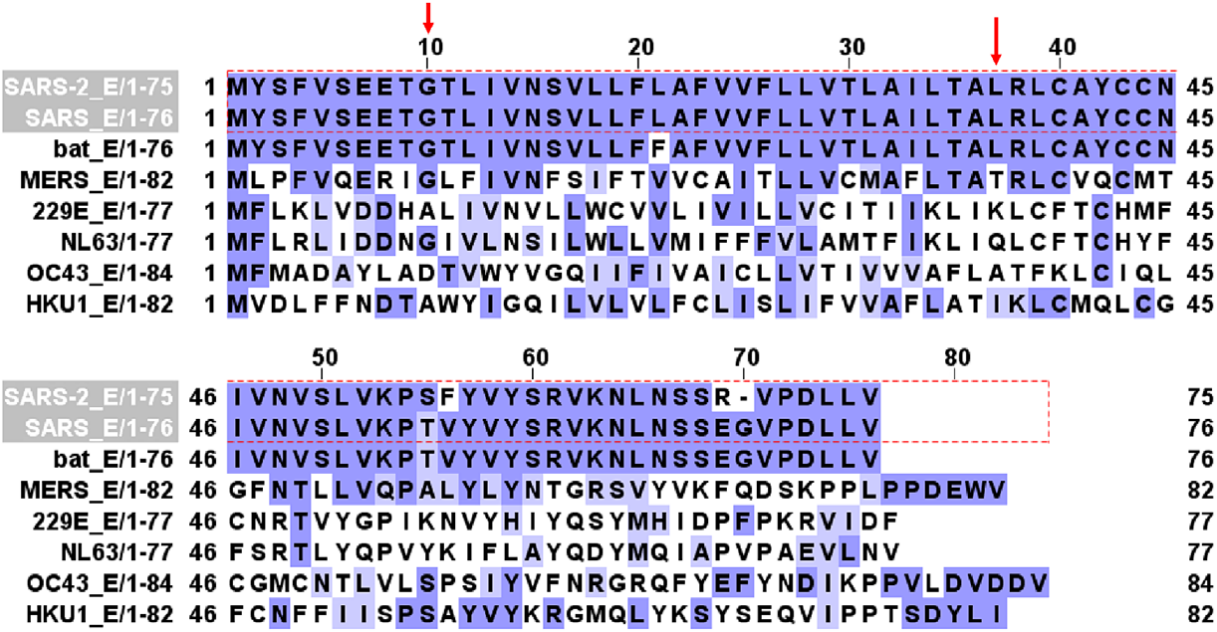
E channel protein sequence alignment in SARS-CoV-2, SARS-CoV, bat SARS-CoV, MERS, 229E, NL63, OC43, and HKU1 coronaviruses. Red arrows mark the beginning (G10) and ending (L37) of the transmembrane domain that forms the channel.

Figure 1 shows E protein sequence alignment in bat SARS-CoV-1, human SARS-CoV-1, human SARS-CoV-2, MERS, and other four common coronaviruses (HCoV-229E, HCoV-NL63, HCoV OC43, and HCoV-HKU1). There is 94.97% homology between SARS-CoV-1 and SARS-CoV-2 E protein sequences. E contains a transmembrane domain between G10 and L37 (red arrows) that forms an ion channel [20,29]. Amino acid residues from G10 to L37 are conserved between SARS-CoV-1 and SARS-CoV-2.

**Fig. 2.**
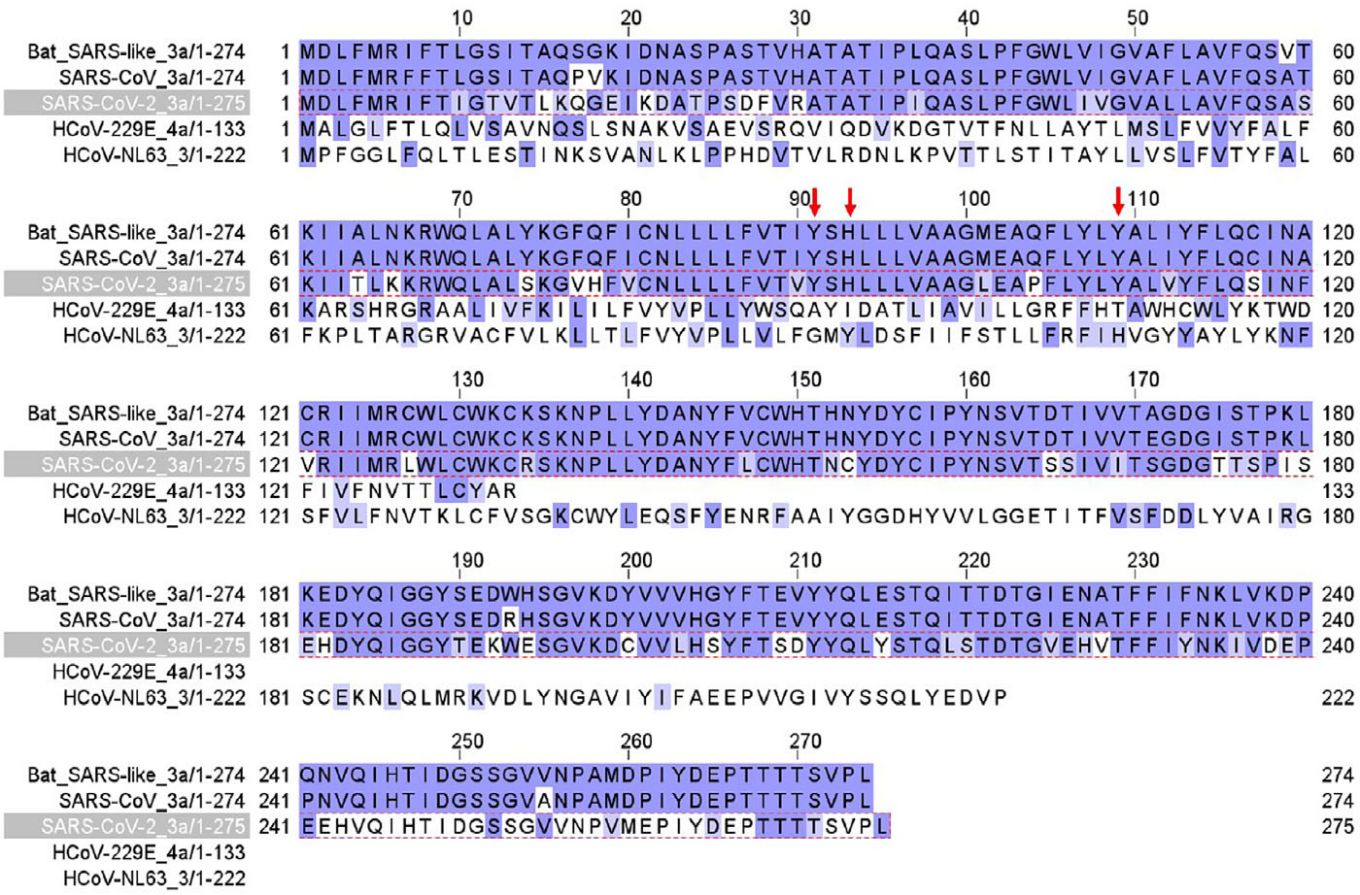
Orf3a channel protein sequence alignment in SARS-CoV-2, SARS-CoV, bat SARS-CoV, HCoV-229E, and HCoV-NL63 coronaviruses. Red arrows label three amino acid residues critical for K+ permeation of the channel.

Figure 2 shows Orf3a protein sequence alignment in bat SARS-CoV-1, human SARS-CoV-1, human SARS-CoV-2, and other two coronaviruses (229E, NL63). There is 73% homology between SARS-CoV-1 and SARS-CoV-2 Orf3a protein sequences. Orf3a contains three transmembrane domains (TD). TD2 and TD3 are required for non-voltage-gated ion channel activity [7]. Three amino acid residues (red arrows), Y91, H93, and Y109, are essential for K^+^ permeability [7] and are conserved in SARS.

### Detection of Orf3a/E Channel Activity in Lentivirus Containing Orf3a and E

**Fig. 3.**
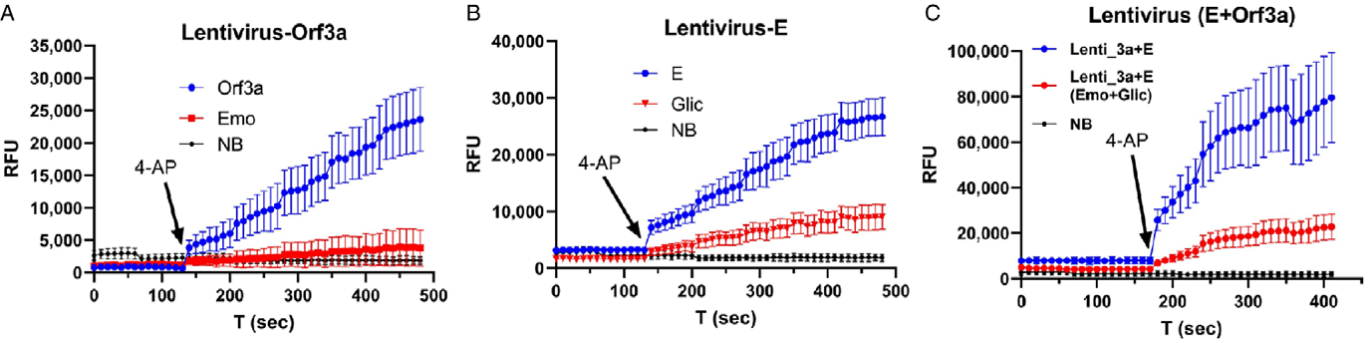
Detection of E/Orf3a channel activity in lentivirus that contain E and Orf3a channels. **A:** lentivirus that contains Orf3a protein. **B:** lentivirus that contains E protein. **C:** lentivirus that contains both Orf3a and E protein. Arrow: time when 4-AP was applied. Emo – emodin; Glic – gliclazide; NB – normal blood sample.

Figure 3 shows a proof-of-concept experiment in a cell-free preparation. Lentivirus containing Orf3a exhibited an increasing fluorescence signal (blue, Fig. 3A) induced by 4-AP (1 mM). In contrast, 4-AP failed to trigger an increase in fluorescence signal in the presence of Orf3a channel blocker, emodin (0.5 mM) (red, Fig. 3A) [25]. Normal blood (NB) from healthy humans did not show a change in fluorescence after 4-AP addition and was used as a negative control (black dot, Fig. 3A).

In lentivirus containing E, 4-AP (1 mM) induced an increase in fluorescence (blue dots, Fig. 3B), which was reduced by 0.5 mM E channel blocker (Glic, gliclazide) [26] (red, Fig. 3B). When both Orf3a and E were used, a significantly larger increasing fluorescence (blue, Fig. 3C) was induced by 4-AP, and this increase was reduced by a combination of 0.5 mM emodin and 0.5 mM gliclazide (red, Fig. 3C). In Fig. 3C, the fluorescence signal intensity is nearly doubled compared to that in Fig. 3A and B, indicating channel activities of both Orf3a and E are additive.

### Detection of Orf3a/E Channel Activity in Blood Samples of COVID-19 Patients

**Fig. 4.**
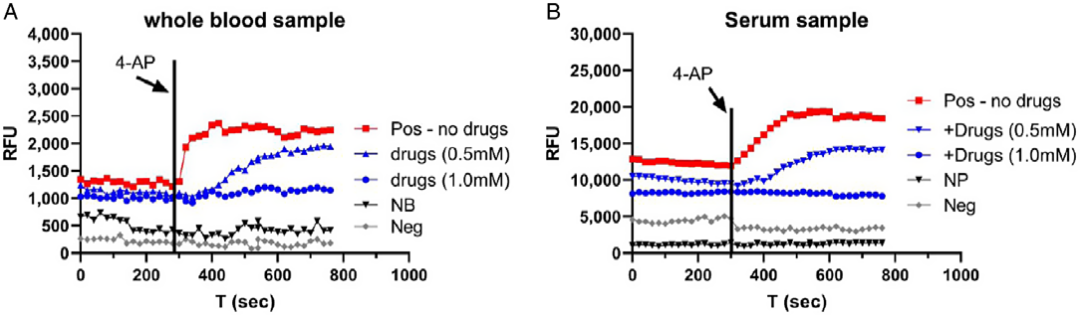
Detection of E/Orf3a channel activity in COVID-19 blood. **A:** whole blood samples. Pos – COVID-19-positive blood; NB – normal blood; Neg – COVID-19-negative blood. **B:** serum sample. Pos – COVID-19-positive serum; NP – normal plasma; Neg – COVID-19-negative serum. Vertical line: time when 4-AP was applied. RFU, relative fluorescence unit.

Figure 4A shows the representative results in a whole blood sample from a COVID-19 antigen test-positive patient. A small volume (5 μL) of sample was used. Fluorescence was induced by 4-AP (black arrow) and caused an increase (red dots), indicating the presence of SARS-CoV-2 virus. This increase in fluorescence was reduced by 0.5 mM emodin and 0.5 mM gliclazide (blue triangle) and blocked by 1 mM emodin and 1 mM gliclazide (blue circle). 4-AP failed to induce an increase in fluorescence in a whole blood sample from a COVID-19 antigen test-negative patient (gray circle) and in normal blood used as a negative control (black triangle).

Figure 4B shows the representative results in a serum sample from a COVID-19 PCR test-positive patient. Fluorescence was induced by 4-AP (black arrow) and caused an increase (red dots), indicating the presence of SARS-CoV-2 virus. This increase in fluorescence was reduced by 0.5 mM emodin and 0.5 mM gliclazide (blue triangle) and blocked by 1 mM emodin and 1 mM gliclazide (blue circle). 4-AP failed to induce an increase in fluorescence in a serum sample from a COVID-19 PCR test-negative patient (gray circle) and in normal plasma used as a negative control (black triangle).

We have tested a total of 42 blood samples. In 18 antigen/PCR-positive samples, our test results found 15 are positive, demonstrating an 83% concordance. In 24 negative antigen/PCR samples, our test results found 21 are negative, showing an 87.5% concordance.

## Discussion

Among the three testing methods, both PCR and antigen tests inform current infection. PCR has extremely high sensitivity (99%) and specificity (97%) [16], serving as an accepted method in diagnostic testing laboratories. The most recent report showed that by targeting three viral genes (Nsp10, NSP12, N), the PCR assays can provide 100% accuracy of test results, based on 23 COVID-19-positive samples and 5 COVID-19-negative samples [5]. Variants that increase the transmission occur mostly in the spike proteins [1]. While a main concern for these variants is the reduced efficacy of vaccine [21] due to decreasing neutralizing activity and immune escape [1,12], there is also a risk that some variants may not be detected by current PCR- and antigen-based testing. Most recently, a new variant B.1.616 has been identified in which only 15% of patients infected with this variant were detected by RT-PCR [14], probably due to nine mutations and one deletion on the spike protein [14]. Antigen test has a higher sensitivity in symptomatic patients (80%) than in asymptomatic patients (41.2%), while its specificity is high (>98%) independent of disease phenotype [23]. Both RT-PCR and antigen tests have not been reported to detect viral proteins in blood.

Recently, a mass-spectrometry-based high-throughput test that targeted proteomics (targeting nuvleoprotein peptides) from “nasopharyngeal and oropharyngeal swabs” was reported [6]. Combined with automation in sample process, this new method has the potential to test large numbers of clinical samples. However, it is unknown whether this method can be applied to blood samples.

In this exploratory research, we targeted Orf3a and E channels for several reasons. First, Orf3a is present only in SARS-CoV-1 and SARS-CoV-2; therefore, a combination of Orf3a and E channel activity is highly selective. Second, highly sensitive detection methods based on fluorescence are used to detect channel activity. Third, utilization of Orf3a and E channel blockers allows us to detect signals only from Orf3a/E while eliminating background fluorescence in blood by subtracting the fluorescence signals in samples without channel blockers. Fourth, protein sequence motifs that control channel activity of Orf3a/E are highly conserved. Thus, detecting the conserved channel activity of Orf3a/E may serve as a valuable tool as viral variants proliferate worldwide. Other interpretations of these preliminary findings are possible.

## Limitations of the Work

The sample size is not large in this work, and samples were collected when disease prevalence was high (approximately up to 50%) in the point-of-care clinic. A larger sample size is needed to test our method for detecting functional viral proteins in blood at lower disease prevalence.

## Conclusions

Our new testing method for SARS-CoV-2 may complement present testing by identifying functional virus in blood samples in a 96-well plate with a total experimental time of around 2 hours. The method can be easily expanded to 384-well plate, and the experimental time can be shortened to less than 1 hour with automation. In addition, our test targets conserved motifs in the Orf3a/E proteins, which are not affected by virus variants. This focus on virally conserved components could alleviate concerns regarding the accuracy of molecular-based methodologies as new variants spread through the community. Finally, detection of the SARS-CoV-2 in blood provides a reasonable explanation as to how the virus spreads from the point of entry in the respiratory tract to other organs including the heart.
